# Chemical and Biological Mobilization of Soil Phosphorus by *ppk*-Expressing Transgenic Rice (*Oryza sativa* L.)

**DOI:** 10.3390/plants15203098

**Published:** 2026-10-10

**Authors:** Shunqing Yang, Mengmeng Wang, Xiaohua Song, Chenyu Wang, Yunfan Zhang, Di He, Jingyun Yin, Xiaotong Ge, Liyu Pan, Minxiang Xu, Yong’an Wei, Liuyan Yang

**Affiliations:** State Key Laboratory of Water Pollution Control and Green Resource Recycling, Key Laboratory of Aquatic Ecosystem Health in the Middle and Lower Reaches of Yangtze River of Ministry of Ecology and Environment, School of Environment, Nanjing University, Nanjing 210023, China; dg1925043@smail.nju.edu.cn (S.Y.); songxiaohua@smail.nju.edu.cn (X.S.); 18851732535@163.com (C.W.); 622025250012@smail.nju.edu.cn (Y.Z.); dg21250011@smail.nju.edu.cn (D.H.); yinjingyun@smail.nju.edu.cn (J.Y.); gxtocean@163.com (X.G.); 602023250035@smail.nju.edu.cn (L.P.); 522024250113@smail.nju.edu.cn (M.X.); 522024250099@smail.nju.edu.cn (Y.W.)

**Keywords:** phosphorus mobilization, *ppk*-expressing transgenic rice (*Oryza sativa* L.), rhizosphere acidification, acid phosphatase, microbial community

## Abstract

Phosphorus (P) availability in alkaline soils is constrained by the immobilization of legacy P, particularly through calcium phosphate precipitation. We used a confined soil–plant microcosm to examine how expression of the polyphosphate kinase gene (*ppk*) is associated with legacy P utilization in rice (*Oryza sativa* L.). We compared *ppk*-expressing transgenic rice (ETR) carrying single-copy (ETRS) and double-copy (ETRD) insertions with their isogenic wild type (WT), without P fertilization. Both transgenic lines had higher root organic acid release and acid phosphatase activity on day 30 and greater plant P acquisition by day 60 than WT; soil pH declined to a greater extent. On day 30, transgenic roots released more oxalate and malate, and their exudates mobilized more soil P than WT exudates in a separate incubation assay. These traits corresponded to greater depletion of the operationally defined HCl-P and NaOH-P pools. Bacterial profiles and PICRUSt2 predictions were descriptive because one pooled library per genotype precluded tests of genotype effects. Diffusive gradients in thin films (DGT) measurements revealed greater rhizosphere labile P in the ETRD early in plant growth, followed by a decline as plant P concentrations increased. This pattern was consistent with an early increase in P supply followed by plant-driven depletion. Net P acquisition in ETRD was 2.55 times that in WT, and ETRD exhibited 53% greater root biomass, while ETRS showed intermediate responses. Collectively, these results link enhanced plant P acquisition to greater P release in exudate incubation assays and higher acid phosphatase activity within this confined system.

## 1. Introduction

Phosphorus (P) is an essential macronutrient involved in photosynthesis, energy transfer, signal transduction, and other physiological and metabolic processes [1]. Soil P availability is often limited by low solubility and strong interactions with soil minerals [2,3]. Phosphate fertilizers are therefore widely applied to sustain crop production. Much of the applied P remains in the soil as poorly available legacy P rather than being taken up by crops. Continued fertilizer use can deplete finite phosphate rock reserves and increase P losses to aquatic ecosystems, contributing to eutrophication [4,5,6,7]. Improving crop access to legacy soil P could reduce fertilizer dependence and conserve P resources. Relevant rhizosphere processes include root-induced changes in soil chemistry, organic acid release, phosphatase activity, and interactions with soil microorganisms [8,9,10].

Rice (*Oryza sativa* L.) supplies food for over half of the global population, making efficient P use important for food production [11,12]. Low soil P availability can nevertheless constrain rice growth and productivity [13]. The *ppk* gene encodes polyphosphate kinase (PPK). Classical microbial PPK1 catalyzes ATP-dependent synthesis of polyphosphate (polyP), which can serve as an intracellular P reserve [14,15]. The *Microcystis aeruginosa ppk* gene was previously introduced into rice by *Agrobacterium*-mediated transformation. Southern blot analysis identified single-copy (ETRS) and double-copy (ETRD) lines [16]. Here, ETR denotes *ppk*-expressing transgenic rice, and S and D denote single- and double-copy insertions. Previous studies reported improved performance under P limitation [16], altered nonstructural-carbohydrate metabolism [17], stronger antioxidant defenses [18], and phosphate-starvation responses in floating-bed systems [19]. How *ppk* expression is linked to organic acid release and whether the trait affects legacy soil P mobilization remain unclear. We therefore used these previously characterized lines to investigate soil processes associated with their reported increase in P acquisition. PPK activity and polyP accumulation were not measured in the present experiment.

Legacy soil P availability depends on interacting chemical, enzymatic, and microbial processes [20,21]. Plants influence these processes by releasing protons, organic acids, phosphatases, and other compounds into the rhizosphere. Organic acids can mobilize mineral-bound P through acidification, ligand exchange, and metal complexation, while phosphatases hydrolyze organic P compounds. Root exudates can also alter microbial communities and may favor groups involved in P transformation [22]. The relative importance of these processes depends on soil properties. Flooding can release P through reductive dissolution of Fe minerals in paddy soils. In alkaline soils, however, legacy P is often held in Ca-associated pools that are less responsive to redox changes [23]. Access to these pools may depend more strongly on root-induced acidification and organic acid release. It remains unknown whether *ppk* expression enhances these processes and improves access to Ca-associated legacy P. Rhizosphere P dynamics may also differ between single- and double-copy lines and warrant further investigation.

We compared ETRS, ETRD, and their isogenic WT in an alkaline-soil microcosm without added P fertilizer to examine legacy P mobilization and acquisition. We measured plant growth and P acquisition, root organic acid release, rhizosphere pH, acid phosphatase activity, and operationally defined soil P fractions. Diffusive gradients in thin films (DGT) measurements were used to quantify rhizosphere labile P availability. We also characterized rhizosphere bacteria to examine whether plant P acquisition was accompanied by differences in the major bacterial groups. These measurements allowed us to assess the chemical and enzymatic responses associated with P acquisition, with bacterial community profiles providing contextual information. The confined system provides a basis for subsequent testing under less restrictive rooting conditions.

## 2. Results

### 2.1. Mobilization of Recalcitrant Phosphorus Fractions and Dynamic P Supply

**Figure 1:**
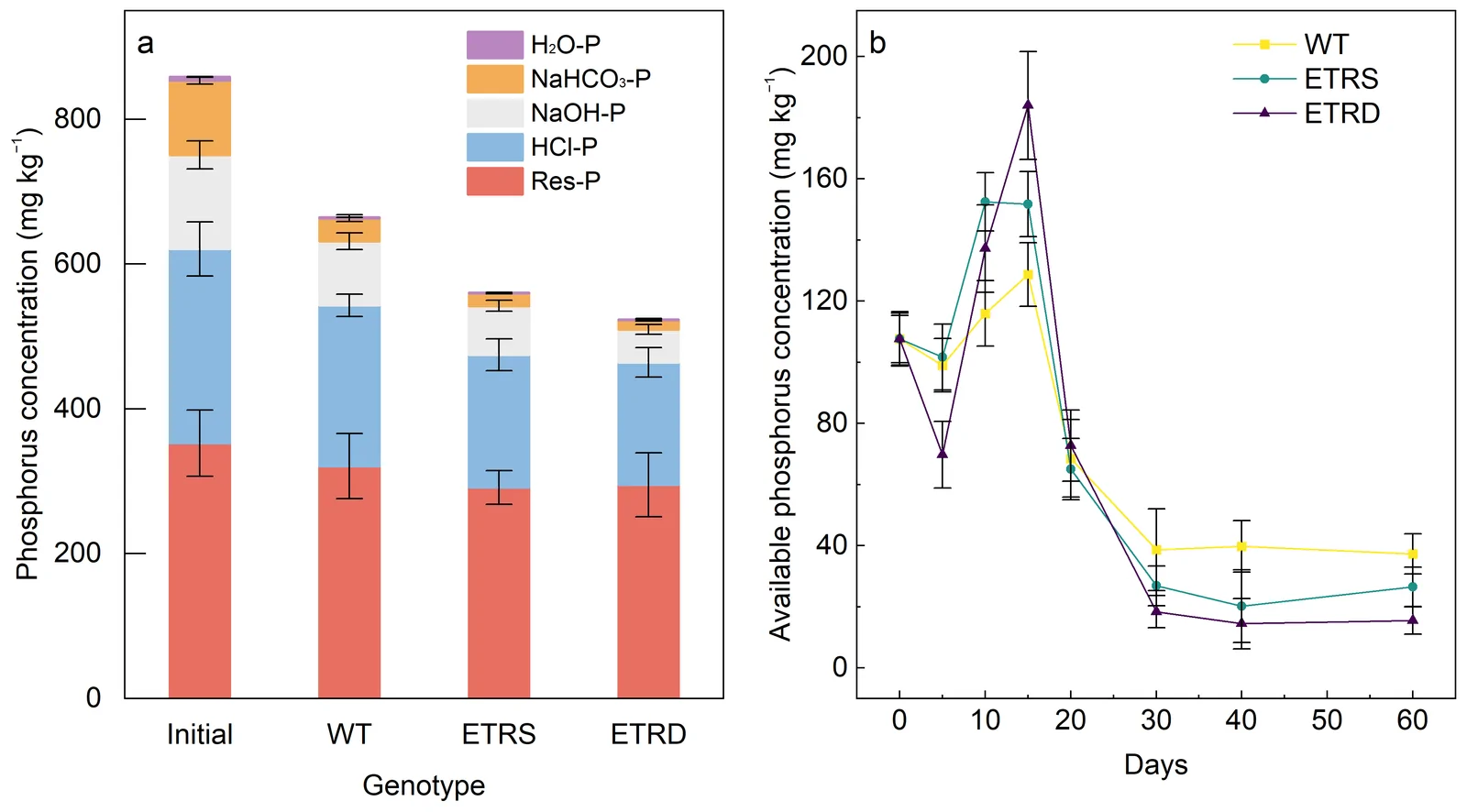
Changes in soil P fractions and Olsen-P concentrations. (**a**) Soil P fractions in the initial homogenized soil and in soils of WT, ETRS, and ETRD at day 60. (**b**) Olsen-P in the initial soil (day 0) and planted soils over time. In panel a, bars show mean fractions and error bars indicate the SD of each individual fraction; initial fractions describe five replicate measurements of the common soil. Planted-soil values are means ± SD (*n* = 5 independent microcosms per genotype at each destructive sampling time); day-0 Olsen-P describes the common initial soil (*n* = 3 replicate measurements). Table S2 reports the day-60 genotype comparisons within each fraction, including significance-letter groupings from one-way ANOVA followed by Duncan’s multiple range test (DMRT, *p* < 0.05) and Welch ANOVA/Games–Howell sensitivity comparisons for NaHCO_3_-P. The common initial soil was excluded from genotype comparisons.

By day 60, soils planted with *ppk*-expressing transgenic rice (ETR) had smaller operationally defined recalcitrant P pools than WT soil (Figure 1a). HCl-P in ETRD soil was 53.1 mg kg^−1^ lower than in WT soil, a reduction of 23.9%, while NaOH-P was 48.2% lower. Hedley fractionation defines extractable pools, not specific mineral phases or transformation pathways. These differences therefore indicate greater depletion of operationally defined Ca-associated and Fe/Al-associated P pools under ETR. The combined difference in HCl-P and NaOH-P relative to WT ranged from 59.6 to 95.8 mg kg^−1^ across the transgenic lines. The smaller H_2_O-P and NaHCO_3_-P pools in ETRD soil at day 60 were consistent with greater plant acquisition of mobilized P. Means, standard deviations, and significance-letter groupings for day-60 genotype comparisons within each fraction are reported in Table S2.

Olsen-P followed different temporal patterns among genotypes (Figure 1b). On day 15, Olsen-P in ETRD soil reached 184.0 mg kg^−1^, or 1.43 times the WT value of 128.7 mg kg^−1^. This early increase was consistent with P mobilization temporarily exceeding plant P uptake from the sampled soil pool. By day 60, Olsen-P in ETRD soil had declined to 15.5 mg kg^−1^, or 41.6% of the WT value (37.3 mg kg^−1^). Together with the higher plant P content, this decline suggests greater P depletion under ETRD. However, concentration measurements alone do not separate gross mobilization from uptake rates.

DGT measurements provide an operational measure of diffusible P supply, including resupply from the soil solid phase. On day 30, DGT-labile P was 3.23, 4.68, and 5.91 mg L^−1^ in WT, ETRS, and ETRD soils, respectively. All three pairwise differences were significant (DMRT, *p* < 0.05; Table 1). By day 60, concentrations were low and did not differ significantly among genotypes. Together with plant P accumulation and soil P depletion, this pattern is consistent with greater early P supply followed by plant-driven drawdown. DGT alone does not distinguish dissolution, diffusion, biological uptake, or other concurrent fluxes.

### 2.2. Acidification and Enzymatic Activity in ETR Soil

We analyzed organic acids in root exudates collected at two growth stages (Table 2). Oxalic, tartaric, malic, and acetic acids predominated, whereas malonic, citric, and succinic acids were present at lower concentrations. The measured organic acid totals in ETR were numerically higher on day 30 than on day 60; no between-time test was performed. On day 30, total release was 66.3 mg g^−1^ root dry weight (RDW) in ETRS and 81.5 mg g^−1^ RDW in ETRD. These values were 1.35 and 1.66 times the WT value (49.0 mg g^−1^ RDW), respectively. The increase was concentrated in specific acids. Oxalic acid release rose from 16.3 mg g^−1^ in WT to 34.0 mg g^−1^ in ETRD, equivalent to 2.09 times the WT value (+17.7 mg g^−1^). Malic acid release rose from 12.4 to 19.4 mg g^−1^ (+7.0 mg g^−1^). Citric and malonic acid release varied less among genotypes.

We assessed P release after adding equal volumes (30 mL) of collected exudate preparations to 5 g of soil in a separate incubation assay. Day-30 preparations yielded net P release of 21.7 ± 2.0, 32.7 ± 4.3, and 41.9 ± 4.2 mg kg^−1^ for WT, ETRS, and ETRD, respectively, relative to the water control. Thus, net P release was 1.51 times the WT value for ETRS and 1.93 times that value for ETRD. These comparisons describe the tested preparations at equal solution volumes, not effects per unit root mass or per unit organic acid. Higher oxalate and malate release offer a plausible explanation, but the assay does not isolate individual compounds or exclude biological contributions.

**Figure 2:**
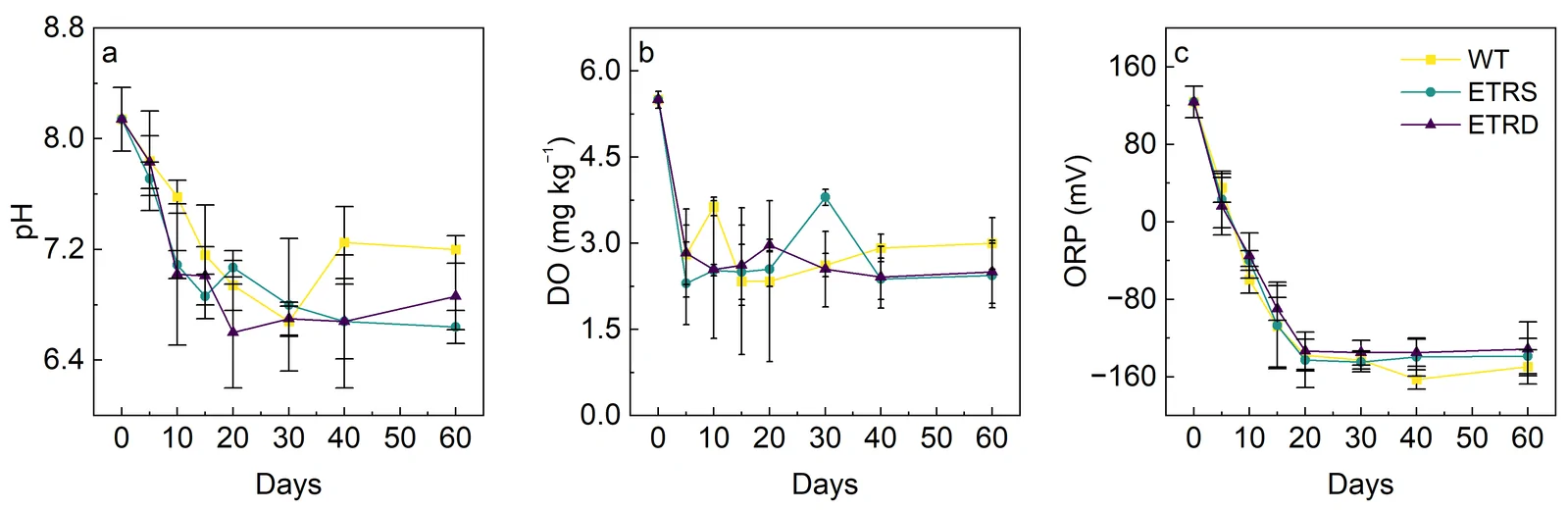
Changes in (**a**) soil pH, (**b**) dissolved oxygen (DO), and (**c**) oxidation–reduction potential (ORP) in soils of WT, ETRS, and ETRD plants over time. The same five replicate microcosms per genotype were measured non-destructively throughout the experiment. Data are presented as means ± SD (*n* = 5).

Greater day-30 organic acid release by ETR coincided with changes in the physicochemical conditions of the bulk root zone. Soil pH declined during days 0–20, with a larger decrease under ETR than under WT (Figure 2a). DO and ORP also declined (Figure 2b,c), indicating oxygen depletion and reducing conditions in the bulk soil. DO and ORP differed little among genotypes during much of the later incubation, and microbial respiration was not measured. These observations therefore do not establish that ETR exudates caused localized hypoxia or reductive Fe dissolution.

We measured acid phosphatase (ACP), alkaline phosphatase (ALP), and phytase activities to assess the enzymatic potential for P mobilization (Table 3). ACP activity was significantly higher in ETR soils than in WT soil, whereas ALP activity did not differ significantly among genotypes. On day 30, ACP activity in ETRS and ETRD soils was 1.10 and 1.70 μmol g^−1^ h^−1^ higher, respectively, than in WT soil. On day 60, the corresponding differences were 3.20 and 3.70 μmol g^−1^ h^−1^, respectively. On day 30, phytase activity was significantly higher in ETRS than in ETRD; WT was intermediate and did not differ significantly from either line. Higher ACP activity indicates greater potential for organic P mineralization in the ETR rhizosphere soils.

### 2.3. Bacterial Community Composition and Predicted Functions Related to P Mobilization

**Figure 3:**
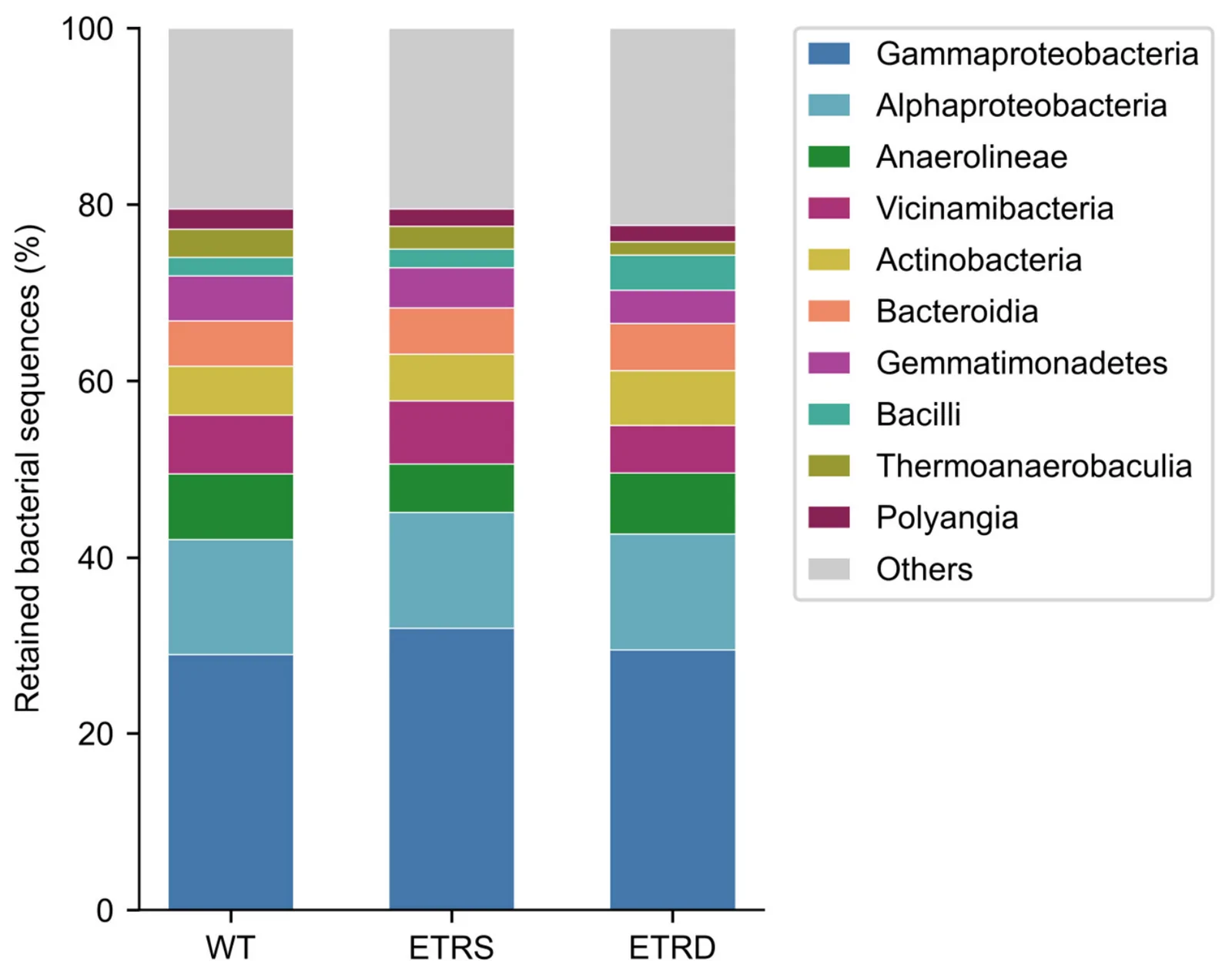
Bacterial class composition in day-60 rhizosphere soil. The same 10 classes, ranked by their combined sequence counts across the three libraries, are displayed for WT, ETRS, and ETRD. Each bar sums to 100% of retained bacterial sequences in the singleton-filtered, non-rarefied ASV table after chloroplast and mitochondrial assignments are excluded (62,254, 67,036, and 88,680 sequences, respectively). Others contain all remaining classes and bacteria unclassified at the class level. Each library was prepared by pooling equal masses of rhizosphere soil from five independent microcosms of one genotype before DNA extraction. There is one library per genotype; no inferential comparisons are shown.

Figure 3 shows the 10 most abundant bacterial classes and Others as percentages of all retained bacterial sequences after singleton filtering and exclusion of chloroplast and mitochondrial assignments. Gammaproteobacteria accounted for 29.02–31.99%, Alphaproteobacteria for 13.00–13.14%, Anaerolineae for 5.49–7.47%, Vicinamibacteria for 5.38–7.16%, and Actinobacteria for 5.25–6.24%. Bacilli accounted for 2.10%, 2.13%, and 3.95% in WT, ETRS, and ETRD, respectively. Others comprised 20.50%, 20.51%, and 22.37%, including remaining classes and bacteria unclassified at class level. These percentages describe the three pooled profiles and do not establish genotype effects or the contribution of any class to P mobilization. Figure S2 shows the corresponding genus-rank taxonomic composition of the same three pooled profiles.

The paired-end FASTQ files contained 329,522 read pairs. After primer trimming and exclusion of read pairs without the required primer matches, 311,959 read pairs entered DADA2, with depths of 90,078–131,179 pairs per library and a median of 90,702. Filtering retained 291,759 pairs and denoising retained 276,088; merging produced 232,513 sequences. Chimera removal retained 220,432 sequences assigned to 8696 amplicon sequence variants (ASVs). Removing features with a total count of one across all three libraries left 218,886 sequences (62,277–89,451 per library; median 67,158) and 7150 ASVs. Rarefaction to 59,163 sequences per library retained a union of 7076 distinct ASVs across the three libraries (Table S1), not the sum of library-level richness.

At this common depth, observed richness was 2860, 2862, and 3651 ASVs in WT, ETRS, and ETRD, respectively. The corresponding Shannon indices were 6.896, 6.946, and 7.142, and Good’s coverage, calculated from the same rarefied table, was 99.890%, 99.814%, and 99.427% (Table S1). Shannon indices were calculated using natural logarithms. Expected-richness rarefaction curves showed diminishing gains with additional sequencing (Figure S1a). Bray–Curtis dissimilarities were 0.403 for WT–ETRS, 0.478 for WT–ETRD, and 0.468 for ETRS–ETRD. The first two PCoA axes explained 60.16% and 39.84% of the variation associated with positive eigenvalues, respectively (Figure S1b). Each genotype was represented by one pooled sequencing library. The additional indices and ordination improve description but do not remedy the lack of independent sequencing replication, nor can they test for genotype effects.

**Figure 4:**
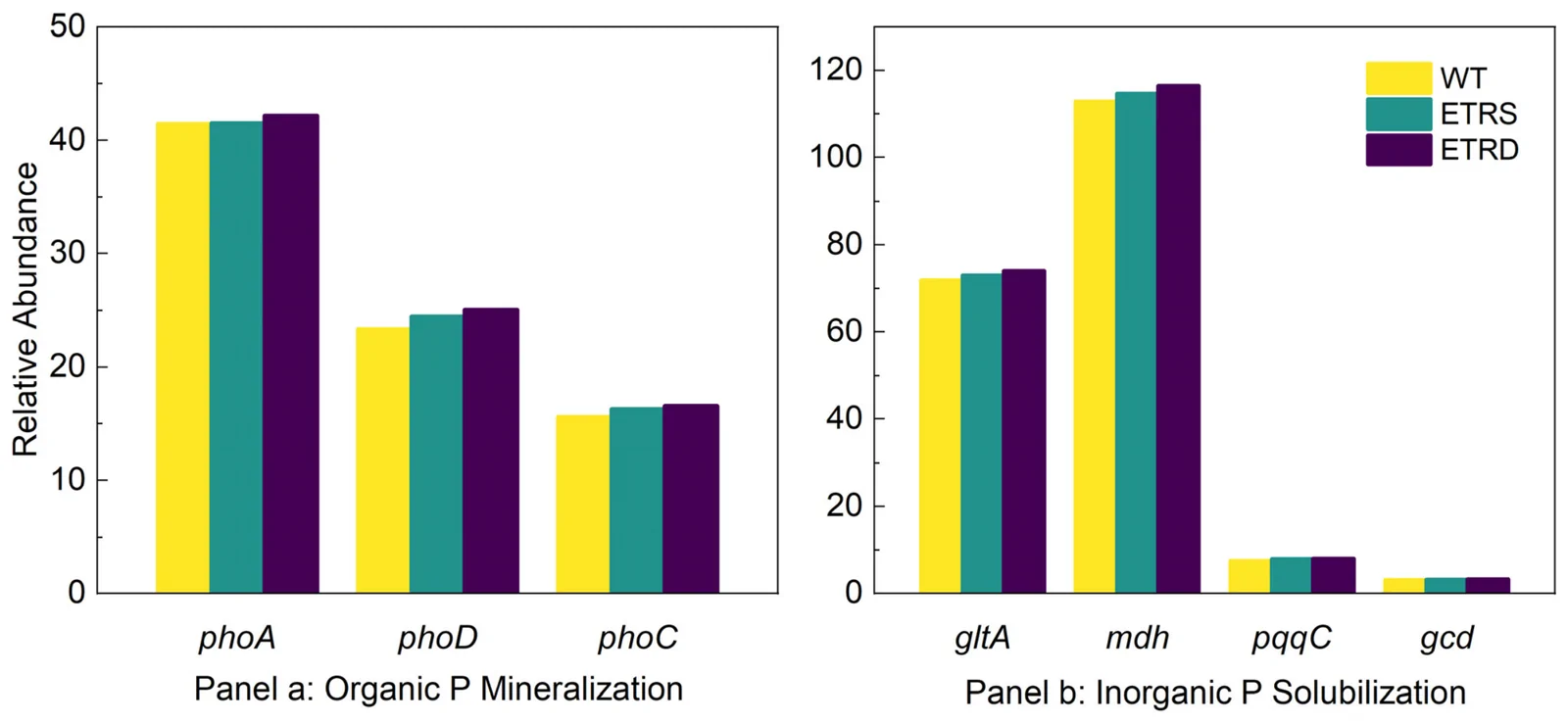
PICRUSt2-predicted bacterial functions related to P turnover in rhizosphere soils of WT, ETRS, and ETRD at day 60. (**a**) Genes annotated for organic P hydrolysis (*phoA*, *phoD*, and *phoC*); (**b**) genes associated with organic acid metabolism (*gltA*, *mdh*, *pqqC*, and *gcd*). Predictions describe genomic potential inferred from 16S rRNA gene profiles, rather than measured gene expression, enzyme production, or P flux.

PICRUSt2 was used to predict gene families related to organic P hydrolysis and organic acid metabolism (Figure 4). These predictions describe genomic potential inferred from 16S rRNA gene profiles, not measured gene expression, enzyme production, or P flux. They do not establish enhanced microbial P mobilization.

### 2.4. Plant Phosphorus Acquisition and Biomass

Initial seedling biomass and P status differed among genotypes before transplantation (Table 4). Net P acquisition accounts for the genotype-specific initial whole-plant P content. Root and shoot biomass, TP concentrations, and whole-plant TP contents are presented in Table 4. Root biomass differed significantly among genotypes and was highest in ETRD. Compared with WT, ETRD exhibited 53% greater root biomass, while ETRS showed a 33.6% increase. Shoot biomass did not vary significantly across genotypes. Enhanced root growth in ETR lines was accompanied by improved plant P acquisition. Net P acquisition was 1.44-fold higher in ETRS and 2.55-fold higher in ETRD relative to WT. Root TP concentrations in ETRS and ETRD were 1.06 and 1.12 times those in WT, respectively. The corresponding shoot TP concentrations were 1.41 and 2.76 times the WT level. These plant phenotypic responses align well with the enhanced rhizosphere P-mobilizing capacity detected in both transgenic ETR lines.

## 3. Discussion

### 3.1. Mobilization of Recalcitrant Legacy Phosphorus and Dynamic Supply

In this confined alkaline-soil system, *ppk*-expressing rice acquired more P and depleted extractable soil P pools more strongly than WT. The initial Olsen-P concentration was 107.6 mg kg^−1^. Accordingly, this experiment examined progressive P depletion in soil that was not initially severely P-deficient. Lower day-60 HCl-P and NaOH-P are consistent with legacy P mobilization, although operational fractions cannot identify the specific minerals supplying P to plants. Rhizosphere P availability reflects the combined effects of dissolution, sorption, biological turnover, and uptake [2,20]. The approximate P inventory in Table S4 uses the nominal initial soil mass and does not establish a closed system-wide P balance.

In ETRD soil, higher early Olsen-P and day-30 DGT-labile P were followed by low day-60 values and greater plant P accumulation. This sequence is consistent with an early increase in available P followed by plant-driven depletion. DGT complements chemical extraction by measuring diffusible P supply and resupply [24]. Neither method, however, can disentangle gross dissolution, readsorption, and uptake rates.

### 3.2. Root-Induced Acidification and Enzymatic P Mobilization

The incubation assay revealed greater P release following addition of ETR exudate preparations, with equal volumes (30 mL) added to 5 g soil. Day-30 ETRD preparations released 1.93 times as much P as WT preparations relative to the water control. Greater oxalate and malate release and lower soil pH are consistent with acidification, ligand exchange, and metal complexation [25,26]. In soils differing in P status, citrate and oxalate additions produced soil- and dose-dependent P responses and also stimulated microbial activity [27]. Our initially P-rich soil therefore represents a context distinct from P-deficient systems, and the response cannot be assumed to persist under low P availability. The assay did not separate chemical release from biological processes or compare effects at equal root mass or organic acid dose.

Declining DO and ORP indicate reducing conditions, which can affect Fe-associated P availability in flooded soils [23]. However, the similar DO and ORP trajectories among genotypes do not identify a reductive pathway specific to ETR. Measurements of Fe speciation and root-surface conditions would help distinguish redox-mediated P release from the effects of organic anions.

Higher ACP activity indicates greater potential for organic P hydrolysis, but its plant or microbial source remains unresolved [20]. Previous work linked *ppk* expression to altered nonstructural carbohydrate metabolism under P deficiency [17]. The higher day-30 organic acid release observed here is compatible with altered carbon allocation, but the present initially P-rich soil differs from those P-deficient conditions. Carbon flux and regulation of organic acid biosynthesis were not measured, so the earlier findings do not establish the molecular cause of exudation in this experiment.

### 3.3. Bacterial Profiles and Their Relationship to P Mobilization

The diversity indices and ordination describe the sequenced bacterial profiles; they cannot overcome the lack of independent replicate libraries or demonstrate selective recruitment by rice genotype. The higher proportion of Bacilli in ETRD is also a descriptive difference among the retained bacterial sequences. Bacilli include P-solubilizing organisms [28], and *Bacillus* isolates have demonstrated extracellular acid phosphatase activity in culture [29]. Nevertheless, class- and genus-level assignments cannot identify the organisms responsible for measured ACP activity or P flux. Previous work examined the relationship between rice purple acid phosphatase 10c and rhizobacterial communities [30]. We measured soil ACP activity without identifying its biological source and profiled bacteria using one pooled library per genotype. These measurements preclude attribution of the observed ACP response to any specific bacterial group.

PICRUSt2 predicts genomic potential from marker-gene profiles [31] rather than measuring transcription or in situ P transformation. These predictions are hypothesis-generating and can guide functional testing, but they do not explain the observed enzyme activities. No fungal community analysis was performed, so this study cannot assess fungal contributions to the observed P mobilization and plant P acquisition. Targeted ITS profiling together with functional assessment would be needed to investigate those contributions.

### 3.4. Implications for Plant P Acquisition and Further Validation

Greater plant P acquisition in this confined system was accompanied by greater P release after exudate addition, higher ACP activity, and greater depletion of extractable soil P pools. The exudate assay supports the contribution from these tested exudate preparations, while the relative chemical and biological contributions and the sources of ACP remain unresolved. Direct comparisons in Table 4 show that ETRD exhibited greater day-60 net P acquisition and root biomass relative to ETRS. These differences apply to the two established lines and do not establish a general gene-copy dosage effect. Genotype-associated differences in seedling biomass and P status were already present before transplantation. Subtracting initial plant P accounts for starting P stocks but cannot remove subsequent effects of initial vigor or nutritional status; seed P reserves and their contribution were not measured separately. Plant transgene expression, PPK activity, polyP accumulation, and root P-transporter activity were not measured in this experiment, so these steps cannot be assigned as the mechanism of the observed responses. Increased P acquisition and root biomass alone do not demonstrate improved agronomic P-use efficiency or yield.

The 50 mL microcosms concentrated root–soil interactions and could alter exudate concentrations, root architecture, and diffusion distances. The observed effect sizes therefore cannot be extrapolated directly to larger soil volumes or field conditions. Further testing should begin with controlled pot experiments across soils and P regimes with progression to field trials contingent on successful pot validation. Pot experiments would enable assessment of P uptake, internal utilization, and P-use efficiency under less constrained rooting environments. Comparisons of sequential extraction with P K-edge XANES have shown that extraction-induced redistribution can bias assignments of mineral P pools [32]. Accordingly, lower HCl-P or NaOH-P here indicates depletion of operational pools, not direct evidence for a specific mineral transformation. Carbon tracing and microbial manipulation could help test the proposed biological links.

## 4. Materials and Methods

### 4.1. Experimental Soil and Design

Soil was collected from a long-cultivated agricultural field in Nanjing (31°48′02″ N, 118°57′10″ E). The soil was air-dried, passed through a 2 mm sieve, and thoroughly homogenized. Initial soil properties were pH 8.14, soil organic matter 29.4 g kg^−1^, total P 858.46 mg kg^−1^, and Olsen-P 107.62 mg kg^−1^ (Table 5). The soil was not artificially alkalized, and no fertilizers were added. Total nitrogen (TN) was measured by the Dumas combustion method using a vario EL III elemental analyzer (Elementar Analysensysteme GmbH, Langenselbold, Germany). Exchangeable potassium was extracted with 1 M neutral ammonium acetate (pH 7.0) and measured using an iCAP 6300 inductively coupled plasma optical emission spectrometer (ICP-OES; Thermo Fisher Scientific, Cambridge, UK). After inorganic carbon was removed with 1 M HCl, soil organic carbon (SOC) was measured by dry combustion using a vario TOC total organic carbon analyzer (Elementar Analysensysteme GmbH, Langenselbold, Germany). Soil organic matter (SOM) was calculated as SOC multiplied by the Van Bemmelen factor of 1.724.

The experiment compared ETRS and ETRD, which carry *ppk* under the CaMV 35S promoter, with isogenic WT rice (*Oryza sativa* L. ssp. *japonica* cv. Nipponbare) [16,33]. In the original construction study, *Microcystis aeruginosa* NIES-843 was selected as the *ppk* donor because it contains chlorophyll a and performs oxygenic photosynthesis, traits shared with higher plants [33]. This rationale does not demonstrate superiority over alternative donors. The lines share the same genetic background apart from the *ppk* transgene insertion. Each sampling cohort included five independent biological replicates per genotype for plant and soil chemical measurements. One planted tube constituted one experimental unit.

A confined microcosm was used to increase the root-to-soil volume ratio and concentrate exudates for observing rhizosphere processes. Healthy two-week-old seedlings at similar developmental stages were transplanted into 50 mL plastic tubes containing 40 g of prepared soil, one seedling per tube. Plants were grown in a controlled-environment chamber with a 12 h light/12 h dark photoperiod at 30 °C during light and 28 °C during darkness. The soil was maintained under continuous flooding throughout the 60-day experiment. Standing-water depth was checked daily and maintained at approximately 5 mm with deionized water, keeping the soil surface covered throughout cultivation. Tube positions were systematically rotated every 5 days to minimize positional effects.

There were 45 independent microcosms per genotype, giving 135 in total. For each genotype, 35 microcosms were assigned to destructive sampling on days 5, 10, 15, 20, 30, 40, and 60 after transplantation. Each sampling cohort contained five independent microcosms. The remaining 10 microcosms per genotype were assigned to separate rhizosphere sampling on days 30 and 60, with five independent microcosms per cohort. No assigned experimental unit was excluded because of plant growth performance.

### 4.2. Soil and Plant Sampling and Processing

The same five microcosms per genotype were monitored non-destructively for soil pH, dissolved oxygen (DO), and redox potential (ORP). Measurements were taken on days 0, 5, 10, 15, 20, 30, 40, and 60. Olsen-P was measured destructively in five independent biological replicates per genotype on days 5, 10, 15, 20, 30, 40, and 60. Day-0 values were replicate measurements of the common homogenized initial soil and were not genotype-specific. Soil P fractionation was performed on the initial homogenized soil and on independent soil samples collected at day 60. Each microcosm was used at only one destructive sampling time.

The five microcosms used for repeated non-destructive monitoring of soil pH, DO, and ORP were harvested as the day-60 destructive-sampling cohort. The day-30 main sampling cohort was used for Olsen-P and DGT determination as well as root-exudate collection. The day-60 cohort was used for Olsen-P measurement, sequential soil-P fractionation, DGT deployment, root-exudate collection, biomass quantification, and plant total-P analysis.

For these chemical measurements, soil from each tube was homogenized and processed separately. Fresh subsamples were reserved for measurements requiring fresh soil, whereas separate subsamples were air-dried for Olsen-P determination and, at day 60, soil P fractionation.

Rhizosphere soil for phosphatase assays was collected from separate sets of five biological replicates per genotype on days 30 and 60 after transplantation. Samples for bacterial community analysis and functional prediction were collected only on day 60. Phosphatase samples from the two dates represented independent experimental units, not repeated measurements of the same plants. Plants were carefully uprooted, and loosely adhering soil was gently shaken off. Soil that remained firmly attached to the root surfaces was then detached using a soft brush and collected as the rhizosphere fraction. All collected samples were immediately flash-frozen in liquid nitrogen and stored at −80 °C until subsequent phosphatase activity assays or DNA extraction, as applicable. Before the enzyme assays, the frozen rhizosphere soil samples were freeze-dried and processed as described in Section 4.4.

After root exudates were collected on day 60, plants were separated into roots and shoots for biomass and P analysis. Both fractions were heated at 105 °C for 30 min and then dried at 65 °C for 48 h to constant weight. Dry biomass was recorded, and the tissues were ground to a fine powder for total P analysis.

### 4.3. Soil Phosphorus Measurements

Soil phosphorus fractions were determined using the sequential extraction procedure described by Hedley et al. [34], as modified by Tiessen and Moir [35]. Briefly, 0.5 g of soil was sequentially extracted with 30 mL each of deionized water, 0.5 M NaHCO_3_ (pH 8.5), 0.1 M NaOH, and 1 M HCl. Each extraction was carried out by shaking the samples for 16 h at 25 °C, followed by centrifugation at 1680× *g* for 10 min and filtration through 0.45 μm membrane filters. The resulting extracts were digested with (NH_4_)_2_S_2_O_8_ in 0.9 M H_2_SO_4_ to oxidize organic phosphorus (OP). Inorganic phosphorus (IP) and total phosphorus (TP) were then quantified, and OP was calculated as the difference between TP and IP.

Residual phosphorus (Res-P) was determined by digesting the final soil residue with concentrated H_2_SO_4_. Phosphorus concentrations in all extracts and digests were measured colorimetrically using the molybdate blue method [36]. Soil available phosphorus (Olsen-P) was extracted by shaking 1 g of air-dried soil with 10 mL of 0.5 M NaHCO_3_ (pH 8.5) for 30 min, followed by filtration. Phosphorus concentrations in the filtrates were measured as described above.

To describe the operational availability of the extracted fractions, three conventional pool categories were used, following Cross and Schlesinger [37] and Negassa and Leinweber [38]: (1) operationally defined labile P pool–the sum of H_2_O-P and NaHCO_3_-P (both inorganic and organic forms), representing the most readily extractable fractions; (2) moderately labile P pool–consisting of NaOH-P (inorganic and organic P), corresponding to P adsorbed on Fe and Al oxides that may be desorbed; (3) relatively stable P pool–calculated as the sum of HCl-P and Residual-P, representing recalcitrant Ca-associated and highly occluded P fractions [35,39].

### 4.4. Rhizosphere Enzyme Activities

Activities of acid phosphatase (ACP) and alkaline phosphatase (ALP) in rhizosphere soil were measured using commercial assay kits (BC0140 for ACP; Solarbio, Beijing, China) following the manufacturer’s instructions. Activities were normalized to freeze-dried soil mass. Briefly, 0.1 g of freeze-dried rhizosphere soil was mixed with 0.05 mL of toluene and allowed to stand for 15 min. Next, 0.4 mL of disodium phenyl phosphate substrate was added. For ACP activity, the assay mixture was incubated at 37 °C for 24 h, whereas for ALP activity, incubation was carried out at 25 °C for 3 h. After incubation, 1 mL of borate buffer was added to terminate the reaction, and absorbance was measured using a UV-1800 spectrophotometer (Shimadzu Corporation, Kyoto, Japan) [40]. In the ACP assay, phenol released from disodium phenyl phosphate was quantified colorimetrically at 660 nm against a phenol standard. ACP activity was expressed as μmol phenol g^−1^ freeze-dried soil h^−1^.

Phytase activity was determined using a separate Solarbio kit. Briefly, 0.1 g of freeze-dried soil was extracted with 1 mL of extraction buffer and incubated at 37 °C for 3 h. Then, 200 μL of molybdenum blue color reagent was added, and the mixture was centrifuged at 10,000× *g* for 5 min at 25 °C. A 300 μL aliquot of the supernatant was mixed with the same color reagent and incubated at 25 °C for 15 min. Absorbance was recorded at 700 nm using the same spectrophotometer [41].

### 4.5. Plant Total Phosphorus Analysis

Total P in plant tissues was measured after sulfuric acid–hydrogen peroxide digestion. Dried, finely ground tissue (0.2 g) was digested with 5 mL of concentrated H_2_SO_4_ and 1 mL of H_2_O_2_ until the solution became clear. The digest was diluted to a known final volume, and total P was measured using the molybdate blue method [42]. Whole-plant P content was obtained from the root and shoot P contents. Net P acquisition was defined as day-60 whole-plant P content minus the initial whole-plant P content of the corresponding genotype, measured in a separate seedling cohort (Table 4).

### 4.6. Root Exudate Collection, Organic Acid Analysis, and Soil Incubation

Root exudates were collected on days 30 and 60 after transplantation. Plants were removed from the tubes, and their roots were washed with tap water followed by deionized water, then gently blotted with absorbent paper. Intact root systems were immersed in 30 mL of sterile water for 4 h, with the shoots above the solution. The collected solution was passed through a 0.22 μm filter and stored at −20 °C for analysis. The roots were then blotted again, and the plants were heated and oven-dried as described in Section 4.2. Organic acid release was normalized to root dry weight at each sampling time.

Organic acids in the exudates were quantified by high-performance liquid chromatography (HPLC) using an Agilent 1200 system (Agilent Technologies, Inc., Santa Clara, CA, USA). The analytes were oxalic, tartaric, malic, malonic, acetic, citric, and succinic acids. Separation was performed on a C18 reverse-phase column using 0.025 M KH_2_PO_4_, adjusted to pH 2.5 with phosphoric acid, as the mobile phase. Detection was performed at 210 nm [43].

To assess P release after exudate addition, 5 g of soil was incubated with 30 mL of exudate solution on an orbital shaker at room temperature for 24 h [27]. The supernatant was collected, and P concentration was measured using the molybdate blue method. Net P release was calculated as the difference between the exudate treatment and the sterile-water control and expressed per kilogram of soil. Equal solution volumes were used; the incubation dose was not normalized to root dry weight or organic acid content. The assay was not designed to separate chemical from biological contributions.

### 4.7. DNA Extraction, Sequencing, and Bioinformatic Analysis

For each genotype, equal masses of day-60 rhizosphere soil from five independent microcosms were combined and thoroughly mixed before DNA extraction and sequencing. This yielded one pooled sequencing library per genotype and three libraries in total. The identifiers were A1 for WT, B1 for ETRS, and C1 for ETRD. The five contributing microcosms did not provide five independent sequencing observations. These pooled libraries were used for bacterial community and functional prediction analyses.

DNA was extracted from 0.5 g rhizosphere soil (stored at −80 °C) using a modified phenol-chloroform extraction protocol. Each sample received 720 μL of sterile phosphate-buffered saline (PBS; 112.87 mM Na_2_HPO_4_, 7.12 mM NaH_2_PO_4_, pH 8.0). We then added 240 μL of TNS buffer (0.5 mM Tris-HCl, 0.1 mM NaCl, 10% SDS, pH 8.0). The mixture was homogenized using a Tissuelyser-48 bead-beating homogenizer (Jingxin, Shanghai, China). The homogenate was centrifuged at 14,000× *g* for 4 min at 4 °C. The supernatant was extracted twice with PCI (phenol-chloroform-isoamyl alcohol, 25:24:1, pH 8.0) and once with CI (chloroform-isoamyl alcohol, 24:1), and the mixture was then centrifuged under the same conditions. Humic substances were removed using DNA-EZ Reagents M Humic acid-Be-Gone B (Sangon Biotech, Shanghai, China). DNA was precipitated from 500 μL of the purified supernatant by adding two volumes of ice-cold ethanol, washed twice with 70% ethanol, air-dried at 37 °C, and finally resuspended in 60 μL of TE buffer.

The bacterial 16S rRNA gene V3–V4 region was amplified with primers 338F (5′-ACTCCTACGGGAGGCAGCA-3′) and 806R (5′-GGACTACHVGGGTWTCTAAT-3′). The forward primer included a sample-specific 7 bp barcode.

PCR was performed in a 25 μL reaction containing 0.25 μL of Q5 High-Fidelity DNA Polymerase (New England Biolabs, Ipswich, MA, USA), 5 μL of 5× Reaction Buffer, 5 μL of 5× High GC Buffer, 2 μL of dNTP mix (10 mM), 2 μL of template DNA, 1 μL each of the forward and reverse primers (10 μM), and 8.75 μL of nuclease-free water.

PCR began with denaturation at 98 °C for 30 s. This was followed by 25–27 cycles at 98 °C for 15 s, 50 °C for 30 s, and 72 °C for 30 s. A final extension was performed at 72 °C for 5 min.

PCR products were examined by 2% agarose gel electrophoresis, and the target amplicons were purified and quantified prior to library preparation. Libraries were prepared using the TruSeq Nano DNA LT Library Prep Kit (Illumina, San Diego, CA, USA). Paired-end sequencing (2 × 250 bp; PE250) was performed on an Illumina NovaSeq 6000 (Illumina, San Diego, CA, USA) using the NovaSeq 6000 SP Reagent Kit (500 cycles) (Illumina, San Diego, CA, USA).

Sequence data were demultiplexed, and primers were removed using cutadapt (https://cutadapt.readthedocs.io/en/stable/). Read pairs without the required primer matches were excluded before DADA2 (https://benjjneb.github.io/dada2/) processing. DADA2 was used for quality filtering, denoising, paired-end merging, and chimera removal. ASV-table filtering and rarefaction were performed in QIIME2 version 2022.11.1 [44]. Features with a total frequency below two across the entire three-library dataset were removed, and each library was rarefied to 59,163 sequences. Observed ASV richness, Shannon diversity (natural logarithms), and Good’s coverage (1 − *f*_1_/*N*, where *f*_1_ is the number of ASVs represented by exactly one sequence within a library after rarefaction and *N* is its rarefied sequence count) were calculated from the rarefied table. Bray–Curtis dissimilarities were visualized using classical principal coordinates analysis (PCoA). Expected-richness rarefaction curves were calculated analytically by sampling without replacement from the filtered counts. These curves assess sampling completeness and do not represent biological replication (Table S1 and Figure S1).

Taxonomy was assigned using the QIIME2 classify-sklearn naïve Bayes classifier with a confidence threshold of 0.7. The classifier used the SILVA 138.1 SSU Ref NR99 reference trained for the amplified region [45]. Figure 3 was calculated from the singleton-filtered, non-rarefied ASV table. ASVs assigned to Bacteria were retained, excluding chloroplast or mitochondrial labels at any taxonomic rank. The denominator was the total retained bacterial sequence count in each library. The 10 classes with the largest combined counts across all three libraries were shown individually; all remaining classes and bacteria unclassified at class level were combined as Others. Figure S2 used the same retained bacterial sequence counts. The 10 most abundant explicit genus-rank annotation labels by combined counts were displayed individually; remaining explicit labels and annotations unresolved at genus rank were grouped separately. This taxonomic display filter was not applied to the diversity calculations in Table S1. With one pooled library per genotype, within-genotype biological variability could not be estimated. Taxonomic profiles, alpha diversity, and beta-diversity ordination were interpreted descriptively. No taxon-specific significance tests, PERMANOVA, differential-abundance tests, or correlation-network inference were applied.

Bacterial functional profiles were predicted from 16S rRNA gene sequences using PICRUSt2 (Phylogenetic Investigation of Communities by Reconstruction of Unobserved States; https://github.com/picrust/picrust2, accessed on 7 October 2026) [31]. Representative sequences were aligned to a reference phylogenetic tree, and the hidden-state prediction algorithm (Castor; https://cran.r-project.org/package=castor, accessed on 7 October 2026) inferred gene-family copy numbers. Predicted functions were mapped to the KEGG (Kyoto Encyclopedia of Genes and Genomes) database to obtain relative abundances of selected functional genes. Targets included genes encoding enzymes for organic P mineralization (*phoA*, *phoD*, and *phoC*). Genes associated with organic acid production and P-solubilization (*gltA*, *mdh*, *pqqC*, and *gcd*) were also examined. Predictions were interpreted descriptively, without inferring gene expression or microbial P flux.

### 4.8. Measurement of DGT-Labile Phosphorus

DGT-labile P was measured using zirconium-oxide DGT probes (Easysensor Ltd., Nanjing, China). Each exposure window was filled with fresh soil paste, and the device was gently tapped to ensure contact between the soil and DGT surface. Probes were deployed for 24 h at 25 °C. P bound to the Zr-oxide gels was eluted with 1.8 mL of 1.0 M NaOH for 24 h. P concentrations in the eluates were measured colorimetrically using the molybdate blue method [24]. The mass of P accumulated in the binding gel (*M*, μg) was calculated using Equation (1):

(1) *M* = *C_e_*(*V_g_* + *V_e_*)/*f_e_*

where *C_e_* corresponds to the concentration of labile P in the eluate, *V_g_* is the volume of the gel, and *f_e_* is the elution efficiency. The *f_e_* value for P is 0.95.

The accumulated P mass was converted to the DGT-measured concentration (*C*_DGT_) using Equation (2):

(2) *C*_DGT_ = *M*Δ*g*/*DAt*

where *A* is the exposed surface area of the DGT device, *D* is the diffusion coefficient of labile P in the diffusive layer, *t* is the deployment time, and Δ*g* is the thickness of the diffusive layer. The diffusion coefficient (*D*) for P at 25 °C was 6.86 × 10^−6^ cm^2^ s^−1^.

### 4.9. Data Analysis

Results are presented as means ± standard deviations (SD). The original non-sequencing analyses used SPSS 26.0 (IBM, Armonk, NY, USA). Overall one-way ANOVA statistics were reconstructed directly from the reported group means, sample SDs, and sample sizes using Python 3.12.14 and SciPy 1.18.1 (Tables S2 and S3). Between-group and within-group sums of squares were calculated from these summary statistics; no replicate values were generated. Reported *p* values are conditional on these inputs and may differ slightly from analyses of unrounded observations. The reported DMRT groupings were checked using the pooled within-group mean square and the studentized-range distribution, with pairwise significance assessed only when the corresponding overall ANOVA was significant. Welch ANOVA and Games–Howell comparisons for NaHCO_3_-P were calculated from the same summary statistics as a sensitivity analysis. These calculations do not verify distributional assumptions from individual observations.

For non-sequencing variables measured at a single sampling time, differences among genotypes were assessed using one-way analysis of variance (ANOVA) followed by Duncan’s multiple range test (DMRT). For variables measured destructively at multiple sampling times, genotype comparisons were performed separately within each sampling time because each time point involved a separate set of independent experimental units.

Soil pH, DO, and ORP were repeatedly measured in the same five experimental units per genotype and are presented descriptively. No genotype, time, or genotype × time effects were inferred for these variables. Statistical significance was set at *p* < 0.05 for analyses in which inferential tests were applied.

The five soil samples contributing to each pooled sequencing library were not treated as five independent sequencing observations.

For destructive plant and soil chemical measurements, one planted tube constituted one independent biological replicate (*n* = 5 per genotype at each sampling time). The summary-statistic calculations retained this experimental sample size and did not treat the three genotype means as three biological replicates. Aliquots or repeated analytical measurements from one tube were treated as subsamples, not additional biological replicates. No biological replicates were excluded from those analyses.

Variables analyzed by ANOVA were not log-transformed or subjected to other variance-stabilizing transformations. Sequence processing, rarefaction, relative-abundance calculation, and functional-abundance normalization are described separately in Section 4.7. An approximate soil–plant P inventory was calculated using 0.040 kg, the nominal initial mass of prepared soil, for both initial and final soil pools. Initial soil P used total P concentration, whereas final soil P used the sum of the reported sequential-extraction fractions. Initial and final whole-plant P contents were then added to the respective soil estimates (Table S4). Final soil dry mass was not measured, and no measured water-phase or other unaccounted P stocks were available; this is an assumption-based inventory, not a closed mass balance.

## 5. Conclusions

In these confined alkaline-soil microcosms without P fertilizer addition, both *ppk*-expressing rice lines acquired more P and produced greater root biomass than WT. Greater day-30 organic acid release and P release after exudate addition, an observed soil pH decline, and higher ACP activity accompanied depletion of extractable soil P pools. The exudate assay supports the contribution from these tested preparations to P mobilization, while the relative chemical and biological contributions, regulation of exudation, and biological sources of ACP remain unresolved.

Bacterial community profiles offer descriptive context but do not constitute evidence for a causal microbial mechanism. Effect sizes, including the 53% greater root biomass in ETRD, are specific to this confined system and cannot be extrapolated directly to field conditions. Further validation should start with controlled pot experiments; field trials should only be pursued upon successful pot-scale validation. Such studies are required to determine whether these observed phenotypic responses enhance agronomic P-use efficiency, grain yield, or reduce crop P fertilizer demand.

## Figures and Tables

**Table 1:** DGT-measured labile phosphorus concentration in soil samples from WT, ETRS, and ETRD plants at days 30 and 60.

Sampling Time	Genotype	*C*_DGT_ (mg L^−1^)
Day 30	WT	3.23 ± 0.47 ^c^
	ETRS	4.68 ± 0.67 ^b^
	ETRD	5.91 ± 0.86 ^a^
Day 60	WT	0.42 ± 0.07 ^a^
	ETRS	0.36 ± 0.05 ^a^
	ETRD	0.38 ± 0.05 ^a^

Data are presented as means ± SD (*n* = 5). Within each sampling time, values followed by different lowercase letters are significantly different among genotypes according to one-way ANOVA followed by Duncan’s multiple range test (DMRT) at *p* < 0.05. Overall ANOVA statistics are reported in Table S3.

**Table 2:** Organic acid composition of root exudates (mg g^−1^ RDW) and net P release after equal-volume exudate addition in the soil incubation assay (mg kg^−1^) for WT, ETRS, and ETRD at days 30 and 60.

Sampling Time	Genotype	Oxalic Acid	Tartaric Acid	Malic Acid	Malonic Acid	Acetic Acid	Citric Acid	Succinic Acid	Total	Net P Release (mg kg^−1^)
Day 30	WT	16.31 ± 2.72 ^c^	7.19 ± 0.71 ^c^	12.40 ± 2.03 ^c^	1.28 ± 0.19 ^b^	7.42 ± 1.10 ^b^	2.05 ± 0.17 ^b^	2.38 ± 0.27 ^b^	49.0 ± 3.7 ^c^	21.7 ± 2.0 ^c^
	ETRS	25.62 ± 3.13 ^b^	9.65 ± 0.96 ^b^	15.42 ± 1.30 ^b^	1.65 ± 0.26 ^a^	8.36 ± 0.80 ^ab^	2.15 ± 0.25 ^b^	3.46 ± 0.51 ^a^	66.3 ± 4.2 ^b^	32.7 ± 4.3 ^b^
	ETRD	34.0 ± 3.21 ^a^	11.38 ± 0.93 ^a^	19.41 ± 2.72 ^a^	1.35 ± 0.17 ^b^	9.72 ± 1.10 ^a^	2.57 ± 0.35 ^a^	3.10 ± 0.49 ^a^	81.5 ± 5.6 ^a^	41.9 ± 4.2 ^a^
Day 60	WT	10.70 ± 1.71 ^a^	7.41 ± 1.12 ^a^	9.62 ± 1.34 ^a^	0.67 ± 0.06 ^a^	1.97 ± 0.29 ^a^	0.76 ± 0.11 ^a^	0.97 ± 0.12 ^a^	32.1 ± 2.82 ^a^	15.7 ± 1.6 ^a^
	ETRS	8.71 ± 1.12 ^b^	6.41 ± 0.70 ^a^	5.77 ± 0.92 ^c^	0.69 ± 0.09 ^a^	1.45 ± 0.17 ^b^	0.51 ± 0.064 ^b^	0.62 ± 0.10 ^b^	24.2 ± 0.99 ^b^	13.7 ± 1.2 ^ab^
	ETRD	6.51 ± 0.59 ^c^	6.85 ± 0.82 ^a^	7.91 ± 1.00 ^b^	0.55 ± 0.09 ^b^	1.43 ± 0.22 ^b^	0.76 ± 0.09 ^a^	0.73 ± 0.11 ^b^	24.7 ± 1.61 ^b^	12.6 ± 2.0 ^b^

Data are presented as means ± SD (*n* = 5). For each variable, statistical comparisons were performed among WT, ETRS, and ETRD within the same sampling time using one-way ANOVA followed by Duncan’s multiple range test (DMRT). Means sharing at least one lowercase letter are not significantly different, whereas means with no letters in common are significantly different at *p* < 0.05. Overall ANOVA statistics are reported in Table S3.

**Table 3:** Activities of acid phosphatase (ACP), alkaline phosphatase (ALP), and phytase in rhizosphere soils of WT, ETRS, and ETRD plants at days 30 and 60.

Genotype	ACP (μmol g^−1^ h^−1^)		ALP (μmol g^−1^ h^−1^)		Phytase (μmol g^−1^ h^−1^)	
Sampling Time	Day 30	Day 60	Day 30	Day 60	Day 30	Day 60
WT	2.20 ± 0.45 ^c^	3.80 ± 0.23 ^c^	0.12 ± 0.04 ^a^	0.26 ± 0.06 ^a^	0.15 ± 0.02 ^ab^	0.09 ± 0.02 ^a^
ETRS	3.30 ± 0.38 ^b^	7.00 ± 0.34 ^b^	0.10 ± 0.04 ^a^	0.22 ± 0.07 ^a^	0.17 ± 0.02 ^a^	0.08 ± 0.02 ^a^
ETRD	3.90 ± 0.35 ^a^	7.50 ± 0.33 ^a^	0.11 ± 0.03 ^a^	0.24 ± 0.05 ^a^	0.13 ± 0.02 ^b^	0.07 ± 0.01 ^a^

Activities are expressed per gram of freeze-dried soil per hour. Data are presented as means ± SD (*n* = 5). For each enzyme and sampling time, means sharing at least one lowercase letter are not significantly different among genotypes; means with no letters in common differ significantly according to one-way ANOVA followed by Duncan’s multiple range test (DMRT) at *p* < 0.05. Overall ANOVA statistics are reported in Table S3.

**Table 4:** Initial and day-60 biomass, total phosphorus (TP) concentration, and whole-plant TP content in WT, ETRS, and ETRD plants.

Genotype		WT	ETRS	ETRD
Dry weight (g)	Initial	0.153 ± 0.004 ^b^	0.154 ± 0.008 ^b^	0.166 ± 0.003 ^a^
	Root	0.217 ± 0.011 ^c^	0.290 ± 0.013 ^b^	0.332 ± 0.027 ^a^
	Shoot	1.936 ± 0.043 ^a^	1.978 ± 0.057 ^a^	1.860 ± 0.111 ^a^
TP concentration (mg g^−1^ DW)	Initial	1.05 ± 0.07 ^c^	1.59 ± 0.13 ^b^	2.00 ± 0.11 ^a^
	Root	3.02 ± 0.18 ^b^	3.21 ± 0.12 ^ab^	3.38 ± 0.19 ^a^
	Shoot	2.37 ± 0.12 ^c^	3.35 ± 0.09 ^b^	6.53 ± 0.24 ^a^
TP content per plant (mg)	Initial	0.16 ± 0.01 ^c^	0.24 ± 0.02 ^b^	0.33 ± 0.02 ^a^
	Whole plant	5.24 ± 0.26 ^c^	7.56 ± 0.27 ^b^	13.27 ± 0.84 ^a^
	Net P acquisition	5.08 ± 0.26 ^c^	7.31 ± 0.27 ^b^	12.94 ± 0.84 ^a^

Data are presented as means ± SD (*n* = 5). Within each measured variable, statistical comparisons were performed among WT, ETRS, and ETRD using one-way ANOVA followed by Duncan’s multiple range test (DMRT). Means sharing at least one lowercase letter are not significantly different, whereas means with no letters in common are significantly different at *p* < 0.05. The term “Initial” refers to the whole-plant status (i.e., the sum of root and shoot) of rice (*Oryza sativa* L.) seedlings prior to transplanting on day 0. Overall ANOVA statistics are reported in Table S3. Initial and day-60 measurements were obtained from separate seedling cohorts, not paired measurements of the same plants.

**Table 5:** Initial physicochemical properties of the experimental soil.

Status	pH	SOM (g kg^−1^)	TN (g kg^−1^)	TP (mg kg^−1^)	Olsen-P (mg kg^−1^)	Exchangeable Potassium (mg kg^−1^)
Initial	8.14 ± 0.20	29.41 ± 0.62	1.97 ± 0.07	858 ± 63	107.6 ± 1.7	56.2 ± 2.9

Data are presented as means ± SD (*n* = 3 replicate measurements). No inferential statistical test was performed because the table describes the initial physicochemical properties of a single homogenized soil.

## Data Availability

Summary measurements and derived diversity results are provided in the article and Supplementary Materials. The raw 16S rRNA gene amplicon sequencing data are publicly available in the NCBI Sequence Read Archive under BioProject accession PRJNA1535796, with Run accessions SRR40871770 (WT), SRR40871769 (ETRS), and SRR40871768 (ETRD). BioSample accessions are SAMN63613530 (A1, WT), SAMN63613531 (B1, ETRS), and SAMN63613532 (C1, ETRD).

## Supplementary Materials

The following supporting information can be downloaded at https://www.mdpi.com/article/10.3390/plants15203098/s1, Table S1: sequencing depth and bacterial diversity; Table S2: day-60 soil P-fraction comparisons; Table S3: overall ANOVA statistics for Table 1, Table 2, Table 3 and Table 4; Table S4: approximate soil–plant P inventory; Figure S1: sampling completeness and descriptive ordination; Figure S2: descriptive genus-rank bacterial composition. The supporting tables and figures are provided in the accompanying Supplementary Materials document.

## Abbreviations

The following abbreviations are used in this manuscript:

*ppk*	polyphosphate kinase gene
ETR	*ppk*-expressing transgenic rice
ETRS	single-copy *ppk*-expressing transgenic rice line
ETRD	double-copy *ppk*-expressing transgenic rice line
DGT	diffusive gradients in thin films
*C*_DGT_	DGT-measured P concentration
RDW	root dry weight
DO	dissolved oxygen
ORP	oxidation-reduction potential (redox potential)
ACP	acid phosphatase
ALP	alkaline phosphatase
NSCs	nonstructural carbohydrates
