# Supplementary material for: Chemical and Biological Mobilization of Soil Phosphorus by ppk-Expressing Transgenic Rice (Oryza sativa L.)
Source: Plants (Basel). 2026 Oct 10;15(20):3098. doi: 10.3390/plants15203098 (PMC13709123; doi:10.3390/plants15203098)
Supplement: Supplementary file 1 [file plants-15-03098-s001.zip › plants-4564463-supplementary.pdf]

Supplementary Materials

Chemical and Biological Mobilization of Soil Phosphorus by *ppk*-Expressing Transgenic Rice

Sampling design: A1 = WT, B1 = ETRS, and C1 = ETRD. Equal masses of day-60 rhizosphere soil from five independent microcosms per genotype were pooled before DNA extraction. This yielded one pooled sequencing library per genotype and three libraries in total. The additional diversity indices and ordination describe these libraries; they do not remedy the lack of independent sequencing replication or test genotype effects. BioProject accession: PRJNA1535796. BioSample accessions: SAMN63613530 (A1), SAMN63613531 (B1), and SAMN63613532 (C1). The raw amplicon sequencing data are publicly available in the NCBI SRA under Run accessions SRR40871770 (A1, WT), SRR40871769 (B1, ETRS), and SRR40871768 (C1, ETRD).

Table S1. Sequencing depth and bacterial diversity

A. Sequencing and processing counts. A1 = WT, B1 = ETRS, and C1 = ETRD. Counts before merging, including FASTQ totals, are read pairs; counts after merging are merged sequences. The DADA2-input minimum, median, and maximum are 90,078, 90,702, and 131,179 pairs, respectively; they are not raw-file or final retained depths. Raw FASTQ exceeds DADA2 input after primer processing by 4,161, 3,667, and 9,735 pairs for A1, B1, and C1 (17,563 in total, 5.33% of raw pairs). These differences were calculated by subtracting the DADA2 input counts from the raw FASTQ read-pair counts. The sequencing service provider confirmed that primer trimming and exclusion of read pairs without the required primer matches preceded DADA2 processing.

Stage	WT	ETRS	ETRD	Total	Min	Median	Max
FASTQ read pairs	94,239	94,369	140,914	329,522	94,239	94,369	140,914
DADA2 input after primer processing	90,078	90,702	131,179	311,959	90,078	90,702	131,179
Filtered	83,997	84,363	123,399	291,759	83,997	84,363	123,399
Denoised	78,945	79,565	117,578	276,088	78,945	79,565	117,578
Merged	66,087	69,677	96,749	232,513	66,087	69,677	96,749
Non-chimeric	62,689	67,515	90,228	220,432	62,689	67,515	90,228
Non-singleton	62,277	67,158	89,451	218,886	62,277	67,158	89,451
Rarefied	59,163	59,163	59,163	177,489	59,163	59,163	59,163

B. Diversity calculated from the ASV table rarefied without replacement to 59,163 sequences per library. Singleton filtering removed features with a total count below two across the entire three-library dataset. The numbers 8,696 before filtering, 7,150 after filtering, and 7,076 after rarefaction are unions of distinct ASVs across the libraries, not sums of library-level richness. Diversity values below use the existing rarefied table, before the additional organelle exclusions applied only to the taxonomic display in Figure 3.

Genotype	Sequences	Observed ASVs	Shannon (ln)	Good's coverage (%)
WT	59,163	2,860	6.896	99.890
ETRS	59,163	2,862	6.946	99.814
ETRD	59,163	3,651	7.142	99.427

Richness, Shannon diversity (natural logarithms), and Good's coverage were all calculated from the same table rarefied to 59,163 sequences per library. Good's coverage was $1 - f_1/N$, where f_1 is the number of ASVs represented by one sequence within the rarefied library (65, 110, and 339 for A1, B1, and C1) and N = 59,163. Library-level singletons can be present after dataset-wide singleton filtering and rarefaction. Coverage describes these sequenced libraries, does not demonstrate complete capture of the bacterial community, and cannot replace biological replication.

Table S2. Day-60 soil P-fraction comparisons

Day-60 genotype comparisons for the P fractions shown in Figure 1a. Values are means ± SD (mg kg^{-1}; n = 5 independent planted tubes per genotype). One-way ANOVA compared WT, ETRS, and ETRD; the common initial soil was excluded. Different letters within a row indicate significant differences according to Duncan's multiple range test (DMRT) at $P < 0.05$.

Fraction	WT	ETRS	ETRD	F (2,12)	*P*
Res-P	321.03 ± 44.94 a	291.57 ± 23.33 a	295.27 ± 44.29 a	0.854	0.45
HCl-P	222.00 ± 15.54 a	182.99 ± 21.96 b	168.91 ± 20.27 b	10.000	0.00278
NaOH-P	88.37 ± 11.49 a	67.83 ± 7.46 b	45.74 ± 6.86 c	29.046	2.52e-05
$NaHCO_3$-P	32.12 ± 4.50 a	17.53 ± 1.05 b	13.15 ± 1.71 c	60.955	5.18e-07
H_2O-P	0.82 ± 0.05 a	0.39 ± 0.05 b	0.39 ± 0.04 b	140.076	4.8e-09

Sensitivity analysis for $NaHCO_3$-P: Welch ANOVA reconstructed from the reported means, SDs, and n gave F = 37.832 and P = 0.0002. Games–Howell P values were 0.00324 (WT–ETRS), 0.000648 (WT–ETRD), and 0.00508 (ETRS–ETRD). All three pairwise differences remained significant. This analysis evaluates sensitivity to the equal-variance assumption; no test of individual-observation distributions was performed for this reconstruction.

ANOVA and sensitivity statistics were reconstructed from the reported means, sample SDs, and n = 5 per genotype. Rounding of the input summaries limits numerical precision. DMRT groupings were checked against the summary-derived pooled within-group mean square. These calculations do not independently verify the underlying individual observations.

Table S3. Overall ANOVA reconstructed from reported summary statistics

Each comparison includes WT, ETRS, and ETRD, with n = 5 independent experimental units per genotype. Tests are separate for each time and variable. F has 2 and 12 degrees of freedom. Calculations use the means and sample SDs reported in main Tables 1–4, without generating replicate values. P values describe overall genotype comparisons, not individual pairwise contrasts. Their precision is limited by rounding of the input summaries. Main-table significance letters describe DMRT comparisons.

Main table	Time	Variable	F (2,12)	P
1	d 30	DGT-labile P	19.153	0.000184
1	d 60	DGT-labile P	1.414	0.281
2	d 30	Oxalic acid	42.713	3.49e-06
2	d 30	Tartaric acid	29.032	2.52e-05
2	d 30	Malic acid	14.039	0.00072
2	d 30	Malonic acid	4.370	0.0375
2	d 30	Acetic acid	6.555	0.0119
2	d 30	Citric acid	5.339	0.022
2	d 30	Succinic acid	7.915	0.00643
2	d 30	Total organic acids	63.271	4.22e-07
2	d 30	Net P release	38.231	6.23e-06
2	d 60	Oxalic acid	14.556	0.000618
2	d 60	Tartaric acid	1.559	0.25
2	d 60	Malic acid	15.326	0.000496
2	d 60	Malonic acid	4.343	0.0381
2	d 60	Acetic acid	8.711	0.0046
2	d 60	Citric acid	12.862	0.00104
2	d 60	Succinic acid	13.164	0.000942
2	d 60	Total organic acids	25.472	4.8e-05
2	d 60	Net P release	4.631	0.0323
3	d 30	ACP	23.754	6.72e-05
3	d 60	ACP	217.916	3.7e-10
3	d 30	ALP	0.366	0.701
3	d 60	ALP	0.545	0.593
3	d 30	Phytase	5.000	0.0263

Main table	Time	Variable	F (2,12)	P
3	d 60	Phytase	1.667	0.23
4	d 0	Dry weight / Initial	8.820	0.0044
4	d 60	Dry weight / Root	49.848	1.54e-06
4	d 60	Dry weight / Shoot	3.081	0.0832
4	d 0	TP concentration / Initial	100.457	3.21e-08
4	d 60	TP concentration / Root	5.869	0.0167
4	d 60	TP concentration / Shoot	885.718	9.28e-14
4	d 0	TP content per plant / Initial	120.556	1.14e-08
4	d 60	TP content per plant / Whole plant	302.764	5.38e-11
4	d 60	TP content / Net P acquisition	290.892	6.81e-11

Table S4. Approximate soil–plant P inventory using the nominal initial soil mass

P inventory (mg per microcosm unless stated)	WT	ETRS	ETRD
Initial soil P estimate	34.34	34.34	34.34
Initial whole-plant P	0.16	0.24	0.33
Initial soil + plant P	34.50	34.58	34.67
Day-60 soil P estimate	26.57	22.41	20.94
Day-60 whole-plant P	5.24	7.56	13.27
Day-60 soil + plant P estimate	31.81	29.97	34.21
Final minus initial inventory	-2.68	-4.61	-0.46
Apparent recovery (%)	92.22	86.68	98.67

Soil P amounts were estimated as concentration (mg kg^{-1}) × 0.040 kg. The 40 g value is the nominal initial mass of prepared, air-dried soil, not a measured final dry mass; no moisture-basis correction is available. The initial soil concentration was 858.46 mg kg^{-1} total P (Section 4.1). Final concentrations are the sums of Res-P, HCl-P, NaOH-P, $NaHCO_3$-P, and H_2O-P in Table S2, which use a different analytical basis from the initial total-P measurement. Plant P contents are the reported means in Table 4. Apparent recovery = 100 × final estimated soil-plus-plant P / initial soil-plus-plant P. Water-phase P, possible handling/removal losses, and other unaccounted pools are not assumed to be zero. Differences cannot be assigned to measured P loss or a specific transformation. This calculation is an approximate inventory under a constant nominal soil-mass assumption, not a closed experimental P balance. No SD or significance test is assigned to these composite estimates because paired component-level data and covariances are unavailable.

Figure S1. Sampling completeness and descriptive ordination

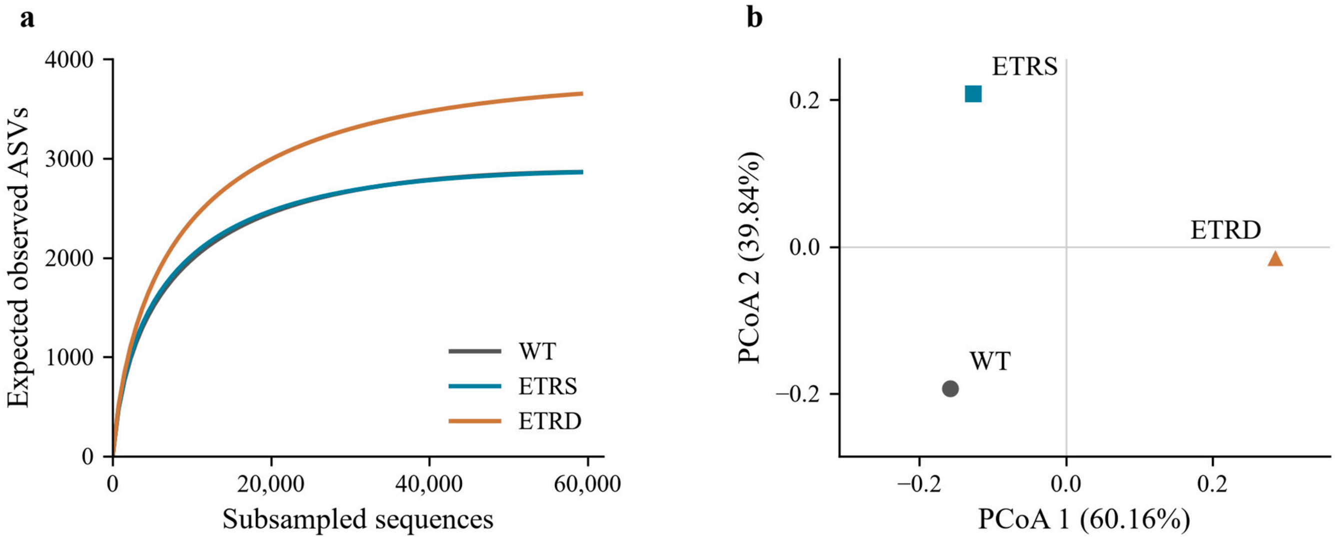

Figure S1. (a) Expected observed ASV richness calculated analytically by sampling without replacement from dataset-wide singleton-filtered, non-rarefied ASV counts at 80 depths from 1 to 59,163 sequences. The curves describe sampling expectations, not biological replicates or confidence intervals. (b) Classical PCoA of Bray–Curtis dissimilarities calculated from the ASV table rarefied to a common depth. Each point represents one library prepared by pooling equal masses of rhizosphere soil from five independent microcosms of one genotype before DNA extraction. With three points, two positive axes account for all variation associated with positive eigenvalues; this geometry does not establish statistically distinct genotype groups. No PERMANOVA or confidence ellipses are shown. Pairwise Bray–Curtis dissimilarities are 0.40307 (WT–ETRS), 0.47804 (WT–ETRD), and 0.46849 (ETRS–ETRD).

Figure S2. Descriptive genus-rank bacterial composition

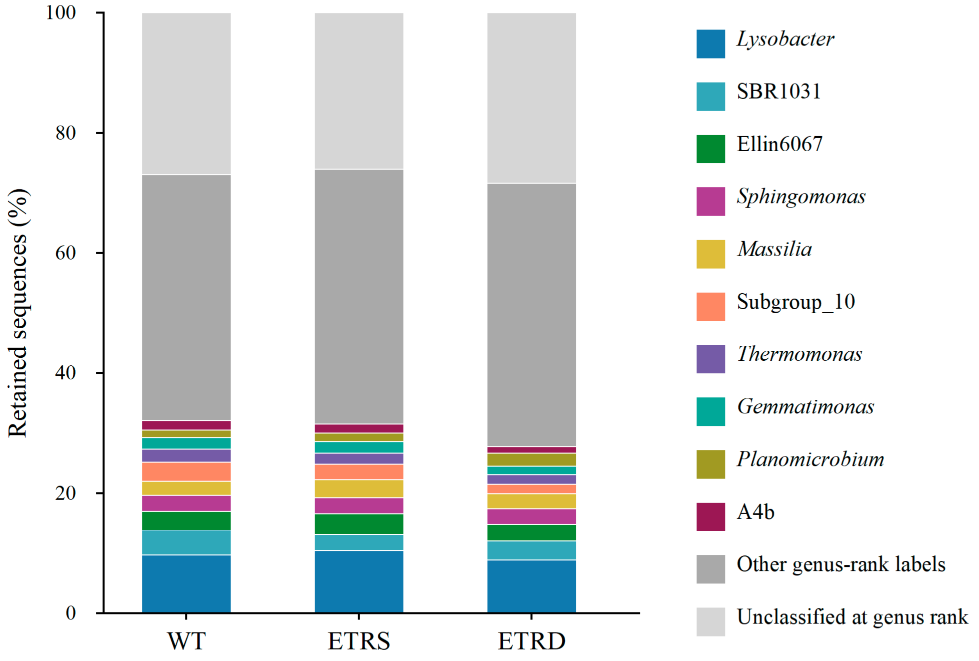

Figure S2. Genus-rank taxonomic composition of day-60 rhizosphere bacteria in WT, ETRS, and ETRD. The 10 explicit genus-rank annotation labels with the largest combined counts across the three libraries are shown individually. Other genus-rank labels contains the remaining explicit labels; Unclassified at genus rank combines blank, uncultured, unclassified, unknown, unassigned, and family-level placeholder annotations. Some explicit labels are provisional database labels rather than formally named genera. Relative abundances use all retained bacterial sequences in the singleton-filtered, non-rarefied ASV table after chloroplast and mitochondrial assignments are excluded (62,254 for WT, 67,036 for ETRS, and 88,680 for ETRD). Each bar represents one sequencing library prepared by pooling equal masses of rhizosphere soil from five independent microcosms before DNA extraction. The comparison is descriptive; no differential-abundance or significance test is shown.
